# Identifying MicroRNA Biomarkers in Idiopathic Intracranial Hypertension: A Pilot Study

**DOI:** 10.1212/WNL.0000000000218162

**Published:** 2026-06-17

**Authors:** Lisa J. Hill, Ghazala Begum, Chloe N. Thomas, Jessica C. Hubbard, Caroline Mugo, Zerin Alimajstorovic, Hannah F. Botfield, Hannah S. Lyons, Mark Thaller, Andreas Yiangou, Christian Ludwig, Olivia Grech, James L. Mitchell, Susan P. Mollan, Alexandra J. Sinclair

**Affiliations:** 1 Department of Biomedical Sciences, School of Infection, Inflammation and Immunology, College of Medicine and Health, University of Birmingham, United Kingdom;; 2 Department of Metabolism and Systems Science, National Institutes for Health Care Research (NIHR) Birmingham Biomedical Research Centre, United Kingdom;; 3 Department of Metabolism and Systems Science, College of Medicine and Health, University of Birmingham, United Kingdom;; 4 Department of Inflammation and Ageing, College of Medicine and Health, University of Birmingham, United Kingdom;; 5 University Hospitals Birmingham NHS Foundation Trust, Queen Elizabeth Hospital, Birmingham, United Kingdom;; 6 Academic Department of Military Rehabilitation, Defense Medical Rehabilitation Centre, Stanford Hall, Loughborough, United Kingdom; and; 7 Ophthalmology Department, Queen's University, Hotel Dieu Hospital, Kingston, Ontario, Canada.

## Abstract

**Background and Objectives:**

Idiopathic intracranial hypertension (IIH) is characterized by raised intracranial pressure (ICP). It predominantly affects women with obesity and can cause disabling headaches and permanent visual loss. IIH is misdiagnosed in up to 40% of cases. Diagnosis and monitoring often rely on frequent invasive lumbar puncture. This pilot study aimed to identify serum microRNA (miRNA) markers associated with a diagnosis of IIH and disease activity and to assess their relevance in the CNS through identification in CSF.

**Methods:**

Serum and CSF samples from participants in the IIH-Weight Trial (a randomized controlled trial comparing the effectiveness of bariatric surgery with that of a community weight management intervention in lowering ICP, recruited 2014–2017) were analyzed at baseline (active disease) and 12 months (participants in remission with no papilledema). Eligible participants were adult women with active IIH (those without papilledema were excluded). Disease activity was assessed by quantifying the degree of papilledema using Spectralis spectral-domain optical coherence tomography. A panel of 40 candidate miRNAs was assessed. Comparator groups included participants with obesity or migraine. Analyses used Student *t* tests and one-way ANOVA followed by Tukey multiple comparisons test. Differentially expressed miRNAs were evaluated in relation to clinical disease activity and metabolic profiles, using linear regression analysis.

**Results:**

Five of 40 serum miRNAs showed significantly lower expression in active IIH (n = 9; 100% female, mean 30.25 ± 4.75 years) compared with IIH in remission. Serum hsa-miR-16-5p had the highest diagnostic performance for active IIH (area under the curve 0.951). Serum hsa-miR-16-5p (*p* < 0.0001; CI −7.286 to −2.503) and hsa-miR-7-5p (*p* = 0.0032; CI −7.240 to −1.372) were significantly lower in active IIH compared with obesity controls (n = 9; 100% female, mean 38.22 ± 7.99 years). Serum hsa-miR-7a-5p were significantly higher in active IIH compared with migraine (n = 12; 100% female, mean 44.67 ± 8.88 years). Only hsa-miR-16-5p differentiated active IIH from both participants with migraine and obesity. hsa-miR-16-5p also differentiated active IIH from IIH remission in CSF (*p* = 0.0354; CI −3.062 to −0.1282). Serum and CSF hsa-miR-16-5p correlated significantly with ICP (serum *p* < 0.0001; CI −0.1660 to −0.0732; CSF *p* = 0.046; CI 0.0018–0.1841), papilledema (serum *p* = 0.0236; CI −0.01548 to −0.0001; CSF *p* = 0.0241; CI 0.002–0.033). hsa-miR-16-5p was associated with metabolites involved in fatty acid metabolism and lipid biosynthesis.

**Discussion:**

Serum hsa-miR-16-5p emerged as a candidate biomarker associated with active IIH. It differentiated active disease from remission, migraine, and obesity. It correlated with clinical and metabolic markers of disease activity. These findings warrant validation in larger studies to assess its potential as a minimally invasive biomarker for IIH diagnosis and monitoring.

**Classification of Evidence:**

This study provides Class III evidence that serum microRNA markers may potentially distinguish active idiopathic intracranial hypertension from remission and controls in adult women.

## Introduction

Idiopathic intracranial hypertension (IIH) is a neurologic disorder characterized by raised intracranial pressure (ICP) and papilledema.^1^ IIH typically affects women of reproductive age with obesity, causing disabling daily headaches which are often migraine-like.^2^ The visual loss can be severe and permanent in up to 25% of cases.^3^ A diagnosis of IIH can be made where there is raised ICP with no underlying cause identified (absence of hydrocephalus, intracranial space-occupying lesions, and normal CSF composition).^4^ IIH was previously considered a rare disease; however, its prevalence is rising rapidly in line with the global obesity epidemic and associated with increased utilization of health care resources. Much of this demand is driven by the need for recurrent hospital visits to monitor the disease activity.^5,6^

IIH has a high misdiagnosis rate (40%) which leads to unnecessary lumbar punctures (LP), drug treatment, and in some cases, surgical procedures.^7^ Currently, LP is required for diagnosis and may also be performed for monitoring ICP.^8^ However, it is an invasive procedure and typically causes distress to patients.^9^ Monitoring disease progression involves evaluating visual function and changes in papilledema.^4^ Papilledema is evaluated using optical coherence tomography (OCT) to quantify retinal nerve fiber layer thickness (RNFL) at the optic nerve head, which can be a challenge for non-ophthalmologists to interpret. Consequently, there is an unmet need for reliable and less invasive methods to diagnose IIH and monitor disease activity, which has been identified by patients and health care professionals as a research priority.^10^ Blood biomarkers represent a promising future alternative. There has been increasing interest around microRNA (miRNA) profiling from blood samples to diagnose and prognosticate cancers and other neurological conditions.^11^

miRNAs are a highly abundant class of conserved noncoding RNA molecules consisting of approximately 22 nucleotides that induce messenger RNA (mRNA) degradation and translational repression. They are ideal biomarkers because they are highly stable in biofluids as they can be transported within protective exosomes.^12^ In addition, miRNAs play a central role in many biological processes including cell metabolism,^13^ apoptosis,^14^ and immune responses.^15^ The role of miRNAs in IIH remains largely unexplored.

The aim of this study was to identify miRNAs in serum that indicate active IIH and evaluate changes in these candidate markers when patients enter remission. The candidate biomarkers were also measured in CSF to gain insights into their relevance to central nervous disease. We compared the IIH candidate serum miRNA biomarkers with participants who have clinical conditions which could be misdiagnosed as IIH (participants with migraine or participants with obesity). Next, we compared these serum and CSF candidate biomarkers against measures of IIH disease activity (ICP and papilledema). Finally, to gain an understanding of the biological relevance of these miRNAs, we determined their association with serum and CSF metabolomic changes (measured by NMR spectroscopy) in participants with IIH. The primary research question for this study was to identify whether serum miRNA markers were able to distinguish active IIH from IIH in remission and from control adult women.

## Methods

### Standard Protocol Approvals, Registrations, and Patient Consents

Adult participants with active IIH were recruited as part of a previously published randomized controlled trial comparing bariatric surgery vs a community weight loss program for the sustained treatment of IIH between 2014-2017 (the IIH:WT Trial; NCT02124486^16^). The results of this study have been previously published.^17^ Briefly, IIH participants were identified from 5 UK centers, and samples were collected following informed, written consent. As part of the IIH:WT trial, non-IIH participants with obesity were recruited through advertisement and written consent was obtained. Ethical approval was received from the National Research Ethics Committee West Midlands-The Black Country REC (14/WM/0011, Dudley, United Kingdom).

Non-IIH, migraine control serum samples were collected under ethical approvals obtained from the Human Biomaterials Resource Centre University of Birmingham under a research license from the HTA (No. 12358) (NRES Committee North West-Haydock; Ref 20/NW/0001). This study was made possible through Application 23-423 under that ethical approval.

This study was conducted in accordance with the principles of the Declaration of Helsinki and approved by the relevant institutional research ethics committees.

### Study Populations

#### Participants With IIH

Eligible participants were female, aged between 18-55, with a body mass index (BMI) ≥35 kg/m^2^, and had active disease (papilledema Frisen grade >1 and raised LP opening pressure ≥25 cmH_2_O) with normal brain imaging (assessed either by magnetic resonance venography or computerized tomography with venography).^8^ Detailed eligibility and exclusion criteria have been published.^16-18^ Exclusion criteria included receiving hormone manipulating medication, significant comorbidities, including known endocrinopathies, and the inability to give informed consent.

Participants with IIH from the IIH:WT Trial attended a baseline trial visit and were randomized to either bariatric surgery or a community weight loss intervention, with follow-up at 12 months.^16^ Blood and CSF samples were collected from those assigned to bariatric surgery (n = 9) at baseline and again at 12 months after surgery (when IIH was in remission defined as LP opening pressure <25 cmCSF). Papilledema was quantified using OCT RNFL (Spectralis SD-OCT, Heidelberg Engineering, Heidelberg, Germany) and assessed by neuro-ophthalmologists at University Hospitals Birmingham NHS Foundation Trust (UHB). LPs were conducted on participants with IIH in the left lateral decubitus position, with knees bent at a 90° angle or more and LP opening pressure was recorded before CSF was collected (up to 15 mL).

Serum was the primary biofluid of interest for biomarker discovery, reflecting the clinical need for a minimally invasive test to support diagnosis and disease monitoring in IIH. CSF analysis was therefore performed as a secondary, mechanistic assessment in participants with IIH only to explore if candidate serum miRNAs showed concordant or divergent expression within the CNS.

#### Serum From Control Participants With Obesity

Eligible control participants with obesity (BMI ≥30 kg/m^2^) and no diagnosis of IIH (n = 9) were recruited at UHB. They met the same inclusion criteria applied to patients with IIH, excluding the presence of IIH or papilledema as confirmed by a neuro-ophthalmologist. Serum collection was performed only at enrolment.

#### Serum From Control Participants With Migraine

Eligible control participants with migraine (and no diagnosis of IIH) (n = 12) were recruited from the tertiary Complex Headache Service at UHB. A diagnosis of migraine was confirmed by a headache specialist according to the International Classification of Headache Disorders 3rd edition (ICHD-3) classification. Serum collection was performed only at enrolment.

#### RNA Extraction and Microarray Analysis to Identify Candidate miRNAs

miRNA was extracted and purified from serum and CSF samples using the miRNeasy Serum/Plasma Kit (Qiagen, UK) following the manufacturer's instructions. miRNA was eluted in RNase-free water. Microarrays were performed by Qiagen using a custom miRCURY LNA miRNA PCR panel (Qiagen, UK) comprising 40 semi-targeted miRNAs selected a priori based on published evidence of differential expression in neurologic disease, traumatic brain injury, ICP regulation, and inflammatory and metabolic pathways relevant to IIH, including miRNAs previously identified in serum and CSF in association with raised ICP.^19,20^ Briefly, isolated miRNA was reverse transcribed using the miRCURY LNA RT kit (Qiagen, UK), diluted 1:40 with RNase-free water, and applied to the custom LNA miRNA PCR panel with targets which were chosen because of their previous association with comorbidities of IIH including obesity, cardiovascular disease, diabetes and androgen excess.

These targets included the following miRNAs: hsa-let-7a-3p, hsa-let-7a-5p, hsa-let-7i-3p, hsa-let-7i-5p, hsa-miR-10b-5p, hsa-miR-143-3p, hsa-miR-143-5p, hsa-miR-145-3p, hsa-miR-145-5p, hsa-miR-148b-3p, hsa-miR-154-3p, hsa-miR-154-5p, hsa-miR-16-1-3p, hsa-miR-16-5p, hsa-miR-187-3p, hsa-miR-190b-5p, hsa-miR-21-3p, hsa-miR-21-5p, hsa-miR-222-3p, hsa-miR-30e-5p, hsa-miR-335-3p, hsa-miR-335-5p, hsa-miR-374b-3p, hsa-miR-374b-5p, hsa-miR-375-3p, hsa-miR-425-5p, hsa-miR-483-5p, hsa-miR-502-3p, hsa-miR-502-5p, hsa-miR-545-3p, hsa-miR-545-5p, hsa-miR-551b-3p, hsa-miR-551b-5p, hsa-miR-589-3p, hsa-miR-589-5p, hsa-miR-7-5p, hsa-miR-9-3p, hsa-miR-9-5p, UniSP3, and UniSp6. The NormFinder algorithm was used to identify stable reference genes to normalize the gene expression to, of which hsa-miR-425-5p, hsa-miR-30e-5p, hsa-miR-148b-3p, and hsa-miR-222-3p were identified.

#### qPCR Analysis of Candidate Biomarkers

To validate the candidate biomarkers identified by the microarray, miRNA was extracted from serum (200 μL) and CSF (500 μL) samples using the same procedure as the microarray analysis (miRNeasy serum/plasma kit; Qiagen, UK). The isolated miRNA was reverse transcribed using the miRCURY LNA RT kit (Qiagen, UK). cDNA was then diluted 1:40 with RNase-free water and added to a qPCR mastermix of miRCURY LNA SYBR Green (Qiagen, UK), RNase-free water, and a primer probe set for hsa-miR-16-5p, hsa-miR-7-5p, hsa-let-7a-5p, hsa-miR-375-3p and hsa-miR-21-5p. Expression levels were normalized to the average of the reference genes for serum hsa-miR-425-5p, hsa-miR-30e-5p, hsa-miR-148b-3p, hsa-miR-222-3p (Qiagen, UK), and the reference gene for CSF hsa-miR-99-5p (as identified from the literature).^21^

### Metabolic Profiling of Serum and CSF Using Proton Nuclear Magnetic Resonance Spectroscopy

We performed an untargeted metabolomic method to identify associations between candidate miRNAs and metabolites which are altered in active IIH compared with remission. Metabolites were extracted from serum and CSF samples as previously described.^22^ In brief, extraction was performed by the addition of methanol and incubation on ice, after which chloroform was added. Vortexing and centrifugation enabled separation of polar metabolites which were then dried and reconstituted in sodium phosphate buffer. A 600 MHz Bruker Avance III spectrometer with a 1.7-mm z-PFG TCI Cryoprobe at 300K was then used to obtain one-dimensional proton NMR spectroscopy (^1^H-NMR) spectra according to the parameters detailed previously.^22^ All NMR spectra were processed using the MetaboLab software.^23^ Probabilistic quotient normalization was used on serum data because of dilution factors. The same did not apply to CSF data because of reduced concentration variation between patients.

### Data Analysis

All analysis and figures were produced using GraphPad Prism version 10 (GraphPad Software Inc, USA). All data were presented as mean and standard deviation (SD). Where appropriate, statistical analyses included Student *t* tests, 1-way ANOVA followed by Tukey's multiple comparisons test, and linear regression analysis. Initial identification of candidate biomarkers, using *t* tests were not corrected for multiple comparisons because this is a hypothesis generating exploratory pilot study. Subsequent analyses did undergo multiple comparison corrections. Linear relationships were assessed using Pearson correlation coefficient (*r*). A receiver operating characteristic (ROC) curve was generated, and sensitivity and specificity values were calculated using the Wilson/Brown method for confidence intervals. Statistical significance is presented as *p* < 0.05. Cq values were obtained from microarray and qPCR experiments. Data were produced for the miRNA of interest, following normalization with the respective reference genes. Data were processed according to Qiagen's customary pipeline which has been previously described.^20^ Consequently, a higher normalized expression value denotes higher levels of the miRNA within the sample.

### Data Availability

Anonymized individual participant data may be made available along with the trial protocol. Proposals should be made to the corresponding author and will be reviewed by the Birmingham Clinical Trials Unit Data Sharing Committee in discussion with the Chief Investigator. A formal Data Sharing Agreement may be required between respective organizations once release of the data is approved and before data can be released.

## Results

### Cohort Characteristics

At baseline, participants with active IIH had an elevated mean ± SD LP opening pressure of 40.08 ± 4.54 cmH_2_0 and the OCT RNFL (papilledema measure) was elevated at 176.36 ± 107.40 µm (Table 1), indicating active papilledema in this cohort at recruitment. On achieving remission at 12 months, the LP opening pressure was significantly reduced (24.94 ± 5.7 cmH_2_0, *p* < 0.0001) and the RNFL papilledema was normalized (97.38 ± 20.99 µm, *p* = 0.0312) (Table 1). Characteristics of the participants with obesity and participants with migraine are described in Table 1. The BMI of the participants with IIH (BMI 40.79 ± 5.83 kg/m^2^) and control participants with obesity (BMI 41.98 ± 5.42 kg/m^2^) were comparable (Table 1). The participants with migraine were not obese (BMI 26.84 ± 8.53 kg/m^2^).

**Table 1:** Description of the Clinical Characteristics of Participants at Baseline With Active IIH and in Remission, Participants With Obesity, or With Migraine

Clinical characteristics of participants	Active IIH (n = 12)	IIH in remission (n = 9)	Participants with obesity (n = 9)	Participants with migraine (n = 12)
Sex (% female)	100	100	100	100
Age, years, mean (SD)	30.25 ± 4.75	—	38.22 ± 7.99	44.67 ± 8.88
BMI, mean kg/m^2^ (SD)	40.79 ± 5.83	30.8 ± 5.82	41.98 ± 5.42	26.84 ± 8.53
Lumbar puncture opening pressure, mean cmH_2_0 (SD)	40.08 ± 4.54	24.94 ± 5.7	—	—
OCT global retinal nerve fiber layer (RNFL) in the left eye, mean µm (SD)	176.36 ± 107.40	97.38 ± 20.99	—	—
Humphrey visual field (24-2) Mean deviation in the left eye, dB (SD)	−4.00 ± 2.93	−3.01 ± 2.22	—	—

Abbreviations: BMI = body mass index; IIH = idiopathic intracranial hypertension.

These data are presented descriptively, and no formal statistical comparisons were performed.

### Candidate Serum miRNA Biomarkers Were Identified by Microarray Analysis

**Figure 1:**
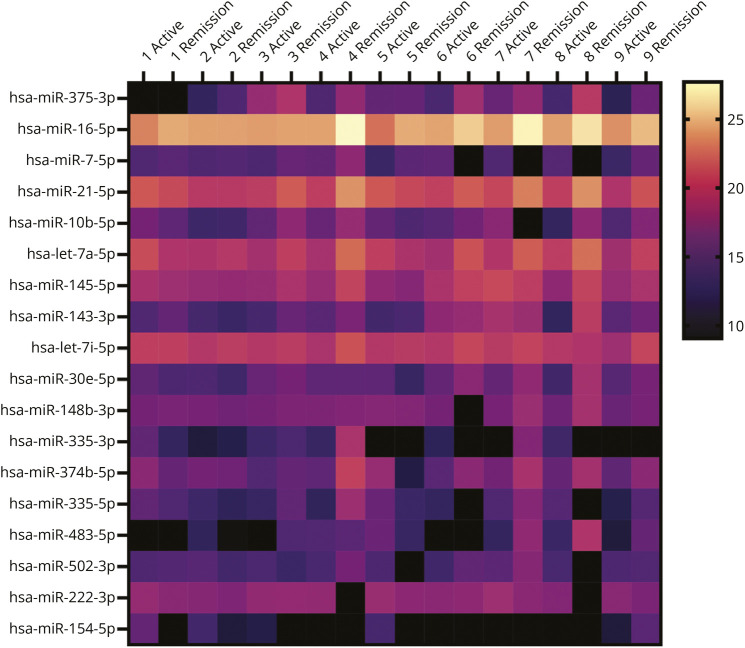
Heatmap Demonstrating the Expression of 18 miRNA in Samples From Participants With Active IIH and When in Remission Data (n = 9, matched pairs) are presented as normalized expression values to reference genes; hsa-miR-425-5p, hsa-miR-30e-5p, hsa-miR-148b-3p, hsa-miR-222-3p. IIH = idiopathic intracranial hypertension; miRNA = microRNA.

A microarray panel profiling 40 different miRNAs was used to identify serum biomarkers in participants with active IIH vs remission. Whilst 40 miRNAs were tested, only 18 miRNAs were found to have stable expression across serum samples (Figure 1).

### Serum miRNA Biomarkers Were Altered Between Active IIH and During Remission

**Figure 2:**
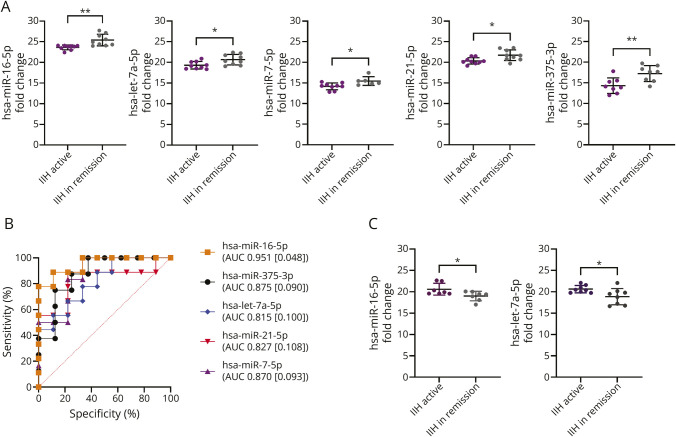
Significant Changes in the Serum and CSF Expression of miRNAs in Participants During Active IIH Compared With Remission (12 Months Later; n = 9 Matched Samples) (A) Five serum miRNAs were significantly different between active IIH and remission. (B) Receiver operating characteristic (ROC) shows the diagnostic potential of the five candidate miRNA biomarkers. hsa-miR-16-5p and hsa-miR-375-3p have the greatest potential as classifiers of a successful intervention. Data are presented as normalized expression values to hsa-miR-425-5p, hsa-miR-30e-5p, hsa-miR-148b-3p, hsa-miR-222-3p with the mean and SD. (C) hsa-miR-16-5p and hsa-let-7a-5p showed statistically lower expression in CSF from IIH participants in remission compared to active disease. Data are presented as normalized expression values to hsa-miR-99-5p with the mean and SD. *p < 0.05, **p < 0.01. IIH = idiopathic intracranial hypertension; miRNA = microRNA.

Five serum miRNAs were significantly altered between participants during active IIH and remission; hsa-miR-16-5p (*p* = 0.0029, CI 0.7037–2.846), hsa-let-7a-5p (*p* = 0.0195, CI 0.2481–2.455), hsa-miR-7-5p (*p* = 0.0205, CI 0.2332–2.351), hsa-miR-21-5p (*p* = 0.0142, CI 0.3199–2.466), and hsa-miR-375-3p (*p* = 0.0082, CI 0.8876–4.975) (Figure 2A). In addition, ROC curve analysis showed hsa-miR-16-5p had the highest level of sensitivity, yielding an area under curve (AUC) of 0.951 (95% CI 0.8573–1.000), suggesting strong discriminatory ability in this exploratory cohort (Figure 2B).

### CSF Expression of Candidate miRNA Biomarkers

To gain insights into the relevance of miRNA biomarkers in the CNS, the 5 candidate miRNA biomarkers identified in serum with the ability to distinguish active IIH were assessed in CSF. We used qPCR to evaluate the candidate markers in the CSF of participants with active IIH and in CSF from these same participants in remission (n = 8). The expression of hsa-miR-16-5p (*p* = 0.0354 CI −3.062 to −0.1282) and hsa-let-7a-5p (*p* = 0.0310 CI −3.345 to −0.1861) were found to be significantly lower in the participants with IIH in remission compared with their active disease states (Figure 2C). miRNAs hsa-miR-7-5p, hsa-miR-21-5p, and hsa-miR-375-3p were not discriminatory (data not shown).

### Serum miRNA Biomarkers Distinguish Between IIH, Obesity, and Participants With Migraine

**Figure 3:**
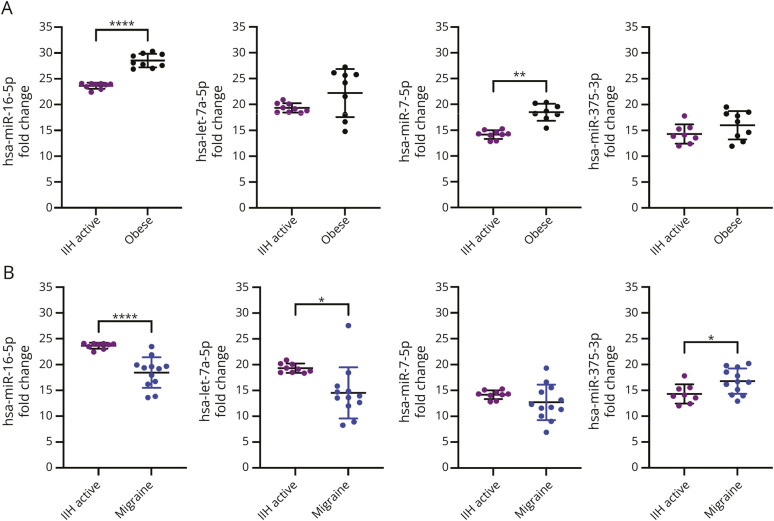
Comparisons of Candidate Serum miRNA Levels Between (A) Active IIH and Obesity and (B) Between Active IIH and Migraine Data are presented as normalized expression values to hsa-miR-425-5p, hsa-miR-30e-5p, hsa-miR-148b-3p, hsa-miR-222-3p with the mean and SD. *p < 0.05, **p < 0.01, ***p < 0.005, ****p < 0.0001. IIH = idiopathic intracranial hypertension; miRNA = microRNA.

Expression levels of the 5 candidate miRNA biomarkers (differentiating active IIH from remission; Figure 2A) were compared with expression levels in participants with obesity or migraine. Expression of 2 miRNAs were found to be significantly lower in the active IIH participants compared with those with obesity (hsa-miR-16-5p, *p* < 0.0001, CI −7.286 to −2.503, Figure 3A, and hsa-miR-7-5p, *p* = 0.0032, CI −7.240 to −1.372, Figure 3A). In comparison, hsa-let-7a-5p and hsa-miR-375-3p demonstrated similar levels of expression in those with active IIH and participants with obesity (Figure 3A).

Participants with migraine had significantly lower levels of expression of hsa-miR-16-5p (*p* < 0.001, CI 2.945–7.420, Figure 3B), hsa-let-7a-5p (*p* < 0.05, CI 0.3209–9.233, Figure 3B), and higher hsa-miR-375-3p (*p* < 0.05, CI −4.922 to −0.0217, Figure 3B) compared with those with active IIH. Hence, only hsa-miR-16-5p expression levels were able to differentiate active IIH from both participants with obesity and participants with migraine. Expression levels of hsa-miR-21-5p was not significantly discriminatory when comparing IIH and non-IIH (obesity and migraine) groups (data not shown).

### Candidate miRNA Expression Levels in Serum and CSF Were Associated With Disease Activity of IIH

**Figure 4:**
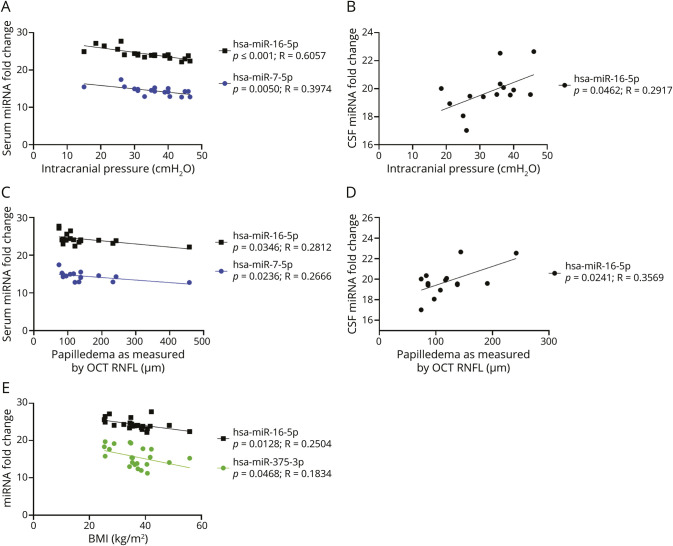
Significant Correlations Identified Between Serum-Derived (n = 18) and CSF-Derived (n = 14) miRNAs With ICP, Papilledema (Measured by OCT RNFL), and BMI (A) In serum, there was a negative correlation between hsa-miR-16-5p and hsa-miR-7-5p and ICP. (B) In the CSF, hsa-miR-16-5p demonstrated a positive correlation with ICP. (C) Papilledema (OCT RNFL) was negatively correlated with serum hsa-miR-16-5p and hsa-miR-7-5p. (D) In the CSF, hsa-miR-16-5p demonstrated a positive correlation with papilledema. (E) Both hsa-miR-16-5p and hsa-miR-375-3p serum levels were significantly correlated with BMI. BMI = body mass index; miRNA = microRNA; OCT = optical coherence tomography; RNFL = retinal nerve fiber layer thickness.

We determined the relationship of the 5 candidate serum miRNA markers to measures of IIH disease activity (LP opening pressure and papilledema). Linear regression analysis revealed that hsa-miR-16-5p expression levels in the serum (*p* < 0.0001, CI −0.1660 to −0.0732, *R*^2^ = 0.6057) and CSF (*p* = 0.046, CI 0.0018–0.1841, *R*^2^ = 0.2917) were associated with ICP (Figure 4, A and B). hsa-miR-16-5p expression levels in the serum (*p* = 0.0236, CI −0.01548 to −0.0001, *R*^2^ = 0.2666) and CSF (*p* = 0.0241, CI 0.002–0.033, *R*^2^ = 0.3569) were also significantly correlated with papilledema (measured by OCT RNFL) (Figure 4, C and D). hsa-miR-7-5p was the only other serum miRNA that was associated with ICP (Figure 4A; *p* = 0.0050, CI −0.1495 to −0.031, *R*^2^ = 0.3974) and papilledema (Figure 4C; *p* = 0.0346, CI −0.0129 to −0.0005, *R*^2^ = 0.2812). hsa-miR-16-5p (*p* = 0.0128, CI −0.1709 to −0.0227, *R*^2^ = 0.2504) and hsa-miR-375-3p (*p* = 0.0468, CI −0.3015 to −0.0023, *R*^2^ = 0.1834) were both significantly associated with BMI (Figure 4E).

### Candidate Serum and CSF miRNAs Are Associated to IIH-Related Metabolites

It has previously been demonstrated that the metabolic profile in the biofluids of patients with IIH is altered.^22,24^ Because miRNAs are key regulators of metabolic pathways, we explored whether there were associations between miRNA expression and metabolite levels in active IIH. Serum hsa-miR-16-5p, hsa-miR-7-5p, hsa-let-7a-5p, and hsa-miR-21-5p were significantly correlated with several serum metabolites (Table 2). In particular, hsa-miR-16-5p, hsa-let-7a-5p, and hsa-miR-21-5p expression levels in the serum were significantly correlated with 2-hydroxybutyrate, creatine, and threonate. Methylmalonate and hsa-miR-16-5p demonstrated the most significant (*p* < 0.0004, CI −0.0955 to −0.03269, *R*^2^ = 0.475).

**Table 2:** Significant Correlations Between miRNA in the Serum and CSF and Metabolite Expression in IIH

miRNA	Serum metabolites	*p* Value	R^2^ value	CSF metabolites	*p* Value	R^2^ value
hsa-miR-16-5p	2-Hydroxybutyrate	0.012	0.279	2-Hydroxyisobutyrate	0.006	0.390
	3-Hydroxyisovalerate	0.050	0.179	3-Methyl-2-oxovalerate	0.011	0.341
	Choline	0.002	0.388	Pyruvate	0.020	0.296
	Creatine	0.007	0.308	Sarcosine	0.013	0.414
	Glutamine	0.036	0.203			
	Isoleucine	0.012	0.275			
	Methylmalonate	<0.001	0.475			
	Succinylacetone	0.016	0.256			
	Threonate	0.005	0.330			
	Valine	0.009	0.298			
hsa-let-7a-5p	2-Hydroxybutyrate	0.006	0.0326	3-Methyl-2-oxovalerate	0.013	0.327
	2-Oxoisocaproate	0.001	0.432	2-Hydroxyisobutyrate	0.022	0.288
	3-Hydroxy-3-methylglutarate	0.027	0.221	Acetone	0.028	0.268
	3-Methyl-2-oxovalerate	0.023	0.234	Alanine	0.022	0.286
	Creatine	0.002	0.394	Isoleucine	0.038	0.243
	Isoleucine	0.011	0.280			
	Propylene glycol	0.012	0.279			
	Succinylacetone	0.025	0.227			
	Threonate	0.017	0.252			
	Valine	0.049	0.180			
	Urea	0.007	0.342			
hsa-miR-21-5p	2-Hydroxybutyrate	0.018	0.249	2-Hydroxyisobutyrate	0.040	0.238
	2-Oxoisocaproate	0.022	0.235	3-Methyl-2-oxovalerate	0.013	0.329
	Creatine	0.003	0.363	Alanine	0.051	0.237
	Threonate	0.006	0.322	Isoleucine	0.033	0.254
	Urea	0.032	0.230	Methylmalonate	0.045	0.228
				Pyroglutamate	0.023	0.861
hsa-miR-7-5p	Choline	0.007	0.353			
	Methylmalonate	0.001	0.506	—	—	—
	Oxypurinol	0.019	0.284			

Expression levels of hsa-miR-16-5p, hsa-let-7a-5p, and hsa-miR-21-5p in CSF were also correlated with CSF metabolite expression (Table 2), with hsa-miR-16-5p and 2-hydroxyisobutyrate being the most significant (*p* = 0.006, CI 0.0017–0.0083, *R*^2^ = 0.390).

### Identified miRNA Markers and Pathways Analysis

**Figure 5:**
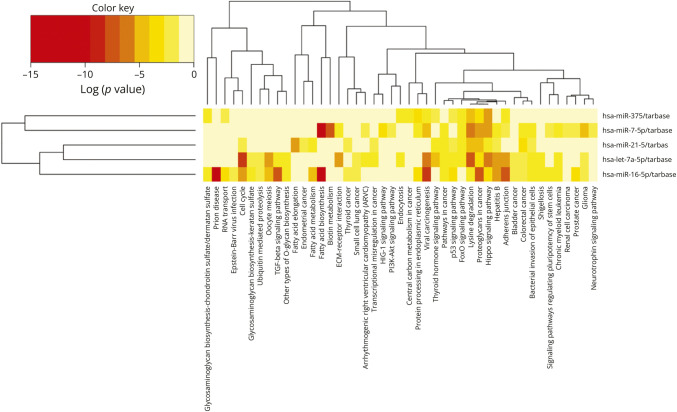
KEGG Analysis Demonstrating the Predicted Pathways that the 5 Candidate miRNAs Have Been Shown to Interact Within

To assess the potential pathways that the candidate markers in this study are working within, KEGG analysis was conducted (Figure 5). Among the networks identified, important pathways for IIH included fatty acid metabolism and fatty acid biosynthesis, which hsa-miR-16-5p and hsa-miR-7-5p were found to be associated with (Figure 5).

### Classification of Evidence

This study provides Class III evidence that serum miRNA markers may potentially distinguish active IIH from remission and from controls in adult women.

## Discussion

In this study, we identified a panel of circulating miRNAs capable of distinguishing active IIH from remission in women. Among these, serum hsa-miR-16-5p emerged as a highly promising biomarker with strong diagnostic and disease-monitoring potential, demonstrating specificity for active IIH and correlation with key clinical markers of disease activity.

Initially, we determined a panel of circulating miRNA biomarkers that were able to discriminate active IIH from remission. Specifically, serum hsa-miR-16-5p, hsa-miR-7-5p, hsa-let-7a-5p, hsa-miR-375-3p, and hsa-miR-21-5p were able to differentiate active IIH, defined by the presence of papilledema (measured by OCT RNFL) and raised ICP (LP ≥ 25cmH_2_O) from IIH in remission (LP < 25cmH_2_O and absence of papilledema). In addition, serum miRNAs hsa-miR-16-5p and hsa-miR-7-5p were able to discriminate between active IIH and participants with obesity, while miRNAs hsa-miR-16-5p and hsa-let-7a-5p could differentiate IIH from participants with migraine. Of these, hsa-miR-16-5p was highly sensitive and specific for diagnosing active IIH (AUC 0.951). In keeping with the serum findings, hsa-miR-16-5p was significantly differentially expressed in the CSF of participants with active disease compared with those in remission. Notably, however, while serum levels of hsa-miR-16-5p were elevated in remission, expression in CSF was reduced, suggesting differential expression patterns centrally vs peripherally during disease remission. The expression of hsa-miR-16-5p also showed a significant correlation with ICP and papilledema (measured by OCT RNFL) severity in both serum and CSF. In addition, hsa-miR-16-5p was significantly associated with metabolic pathways involved in fatty acid metabolism and lipid synthesis, processes implicated in the pathogenesis of IIH.^25^

Emerging research has begun to explore the roles of miRNAs in raised ICP, with early studies suggesting their potential as biomarkers and modulators of disease. Notably, gene expression profiling of exosomal miRNAs in plasma and CSF highlighted miR-9 and miR-16 upregulation in patients with mildly elevated ICP (>20 cmH_2_0) compared with lower ICP states, and these were linked to inflammatory and immune-related pathways.^19^ However, the authors of this study note the limitations of a small sample size of women and that their control groups included people with underlying health conditions which may account for differences noted here. Of note, miR-16-5p is derived from the miR-16-1 gene, which is known to be highly expressed in the brain.^26^

Growing evidence suggests that IIH is a metabolic disorder, rather than solely a condition of the CNS.^1,22,27^ Studies have identified a distinct metabolic profile in IIH patients which was associated with markers of disease activity, indicating dysregulated respiratory chain metabolism (lactate:pyruvate), altered CSF dynamics (urea), and disrupted ketone body metabolism (acetoacetate).^22^ In addition, perturbation in the metabolism of amino acids and lipids including fatty acids have been observed in patients with IIH.^25^ miR-16-5p has been increasingly associated with metabolic disease, especially in relation to insulin resistance and glucose homeostasis in diabetes, suggesting its key role in systemic metabolic dysregulation.^28-30^ Similarly, miR-16 is often grouped with other metabolic miRNAs which are known to modulate lipid metabolism, insulin secretion, and glucose uptake.^13,31,32^ Metabolic dysregulation and insulin resistance are key features of IIH.^27,33^

The miR-15/16 family is highly conserved, widely expressed and has been characterized in oncology as a tumor suppressor, where it can limit proliferation and promote apoptosis through regulation of cell-cycle and prosurvival pathways.^34^ However, altered miR-16-5p expression has also been reported across diverse nonmalignant conditions. These include sepsis-associated acute kidney injury,^35^ IgA nephropathy,^36^ oxidative stress-mediated cardiomyocyte injury,^37^ and COVID-19 severity.^38^ These highlight the context-dependent and pleiotropic roles that may reflect broader inflammatory/stress biology, rather than disease specificity. Importantly, the pleiotropic metabolic roles of miR-16-5p are particularly relevant in the context of IIH, given the strong association between IIH and obesity.

Common (polygenic) obesity arises from the cumulative effects of multiple genetic variants interacting with obesogenic environments and epigenetic mechanisms, including DNA methylation, histone modification, and miRNA-mediated regulation of energy balance and adipose biology.^39^ Given that obesity is the strongest modifiable risk factor for IIH,^39^ these complex genetic-epigenetic pathways may contribute to disease susceptibility and provide a biological rationale for exploring circulating miRNAs as candidate biomarkers.

KEGG analysis in our study showed that hsa-miR-16-5p is involved in fatty acid metabolism and biosynthesis which is understood to be dysregulated in IIH.^25^ Given the known involvement of miR-16-1 in fatty acid metabolism,^40^ and the results of our pathway prediction, hsa-miR-16-5p may also possess mechanistic relevance in IIH pathophysiology, further supporting its potential as a candidate biomarker. Given its links to metabolic pathways, dysregulation of miR-16-5p in our IIH cohort may be indicative of the metabolic dysregulation known to occur in IIH.^41^ hsa-miR-16-5p was also found to be differentially expressed in women with chronic migraine.^42^ In the study by Ornello et al., baseline levels of circulating miRNAs were analyzed to assess their association with treatment response to Erenumab, measured by the change in monthly migraine days from baseline to post-treatment. Patients were classified as low, medium, or high responders based on this change. Interestingly, circulating hsa-miR-16-5p were lower in the medium response group compared with the low and high responders showing a nonlinear pattern with treatment efficacy. In agreement with our results, we observed that hsa-miR-16-5p levels were lower in participants with migraine compared with those with active IIH, indicating a potential diagnostic value in discriminating between these two disorders.

Furthermore, we found that hsa-miR-16-5p expression was increased in remission compared with active disease, suggesting that this miRNA may reflect disease activity. Collectively, these observations support a potential role for this miRNA as a biomarker that may be able to differentiate between headache subtypes and monitor IIH disease activity which would have meaningful impact for delivery of patient care and potentially for research inclusion criteria.^43^ Another meaningful clinical application would be an evaluation of hsa-miR-16-5p expression in people diagnosed with IIH without papilledema.^44^

In exploratory diagnostic developmental studies, it is critical to ensure that control groups are well considered to allow accurate comparisons. As such, one of the key strengths of our investigation was the addition of a non-IIH obese cohort and a non-IIH migraine control group. This enabled us to rule out that miRNA may be influenced by comorbidities of IIH including hsa-miR-375-3p, hsa-let-7a-5p, and hsa-miR-21-5p. Previous studies have shown that these markers such as hsa-miR-375-3p have a strong involvement in obesity.^45^ Furthermore, hsa-let-7a-5p has been extensively studied for inflammation and is identified as a key miRNA in several diseases.^46^ However, investigations have also found these markers to have a role in migraine,^47^ and they similarly were differentially expressed in migraine controls in this study. Therefore, the involvement of these miRNAs in multiple pathways may hinder their use as a diagnostic or monitoring test in IIH.

A limitation of this study is that we assessed a restricted panel of only 40 miRNAs in a small cohort. It is possible that additional informative targets may emerge from broader profiling approaches in future studies. Expanding the panel may allow for a multimarker algorithm for diagnosing or monitoring in IIH. We acknowledge that initial selection of candidate biomarkers did not undergo correction for multiple comparisons and hence, the results are not as statistically robust; however, they still provide initial potentially important findings for future validation. Subsequent analyses were corrected for multiple comparisons, and the results continued to provide significance in respect to miR-16-5p and its involvement in IIH disease activity and differentiation from control groups strengthening confidence in the findings.

The participants in this cohort were all women, and hence, the results are not generalizable to men living with IIH. We acknowledge that optic nerve atrophy, as measured by OCT ganglion cell layer, was not formally included in the linear regression models evaluating associations between papilledema (measured by RNFL OCT) and miRNAs. However, ganglion cell layer analysis in this cohort from the IIH:WT did not demonstrate evidence of atrophy. This is a small exploratory pilot study and the miRNA candidates observed here require validation in larger, independent prospectively collected cohorts taken from a real-world clinical practice consisting of unselected patients being evaluated for consideration of the diagnosis of IIH. Evaluation in the CSF was performed to confirm the biological relevance of this miRNA in the CNS but not to develop a CSF biomarker to be used in clinical practice. Serum is preferable because it is less invasive, but other less invasive biofluids, such as saliva, should also be explored. Salivary miRNA has shown promise in a range of diseases including neurodegenerative disease and migraine.^48-50^ Although this study benefitted from multiple non-IIH control groups, we did not assess candidate miRNA levels in a control group undergoing bariatric surgery. As a result, we cannot definitively attribute the observed changes in miRNA to disease resolution alone because they may also be influenced by the effects of bariatric surgery itself.

In conclusion, our study has identified a panel of miRNAs capable of distinguishing active IIH from remission. Serum hsa-miR-16-5p is the most promising as a diagnostic biomarker, being highly sensitive and specific for diagnosing active IIH and able to differentiate active IIH from both participants with obesity and participants with migraine: conditions which share clinical features akin to IIH. Serum hsa-miR-16-5p is also suitable as a biomarker to monitor disease because it differentiates those participants with active disease from those in remission and importantly correlates with markers of disease activity (ICP and papilledema). Serum hsa-miR-16-5p is likely to be relevant to disease pathogenesis because it was also significantly different in the CSF and highly correlated with metabolic pathways associated with IIH pathogenesis. The next step would be to translate this potential diagnostic and disease monitoring biomarker into clinical practice in IIH. Ideally, hsa-miR-16-5p would be validated in a larger independent real-world cohort containing patients referred for diagnostic evaluation for IIH and tracked longitudinally.

The development of a validated serum biomarker for IIH would represent a meaningful advancement in clinical practice by enhancing diagnostic precision and speed, while simultaneously reducing patient burden by potentially obviating the need for LP. Moreover, if hsa-miR-16-5p is established as a reliable serum biomarker for disease monitoring, it could substantially alleviate the demands of current surveillance strategies, such as frequent visual assessments and episodic lumbar punctures. It also provides potential to introduce evidence-based decision making for the use of pharmacologic therapies. This would not only alleviate the patient's burden but also reduce the substantial healthcare resource utilization currently associated with the management of IIH.

## Glossary

AUC: area under curve
BMI: body mass index
ICP: intracranial pressure
IIH: idiopathic intracranial hypertension
LP: lumbar punctures
miRNA: microRNA
OCT: optical coherence tomography
RNFL: retinal nerve fiber layer thickness
ROC: receiver operating characteristic
